# A pilot study of game-based learning programs for childhood cancer survivors

**DOI:** 10.1186/s12885-022-09359-w

**Published:** 2022-03-29

**Authors:** Daisuke Masumoto, Etsuko Nakagami-Yamaguchi, Misako Nambu, Miho Maeda, Hideko Uryu, Akira Hayakawa, Zayar Linn, Satoshi Okamura, Kosuke Kurihara, Kentaro Kihira, Takao Deguchi, Hiroki Hori

**Affiliations:** 1 Department of Medical Education, Mie University Graduate School of Medicine, 2-174 Edobashi, Tsu, Mie 514-8507 Japan; 2 Department of Medical Quality and Safety Science, Osaka City University Graduate School of Medicine, Osaka, Japan; 3 School of Systems Information Sciences, Future University Hakodate, Hakodate, Japan; 4 Department of Pediatrics, Nippon Medical School, Tokyo, Japan; 5 Department of Pediatrics, Center Hospital of the National Center for Global Health and Medicine, Tokyo, Japan; 6 Department of Palliative Medicine, Yodogawa Christian Hospital, Osaka, Japan; 7 Department of Pediatrics, Mie University Graduate School of Medicine, Tsu, Japan

**Keywords:** Childhood cancer survivors, Patient education, Computer game, Health locus of control

## Abstract

**Background:**

Childhood cancer survivors lacking awareness on their potential risks of late effects often fail to seek adequate follow-up care. Patient education matching their preference is of great importance to improve their adherence to survivorship care. In this study, we developed two age-dependent game-based learning programs, which enable continuous approaches for childhood cancer survivors along their intellectual maturation. Then, we assessed the effectiveness of the programs.

**Methods:**

Childhood cancer survivors over 10 years of age who regularly visited a long-term follow-up clinic were enrolled in this study. They were requested to play either of two different types of game tools, one for school children and another for adolescents and young adults, for one month at home. To evaluate the educational effects of the programs, they were examined for health management awareness, self-esteem, and knowledge on cancer-related late effects before and after the intervention with age-based questionnaires and knowledge tests.

**Results:**

Among 83 participants, 49 (59.0%) completed the assessments over the period of 12 months. The health management awareness and knowledge levels increased significantly at 1-month after the intervention as compared to the baseline in both school children and adolescents/young adults (for health management awareness, *p* = 0.011 in elementary school children; *p* = 0.007 in junior high school children; *p* < 0.001 in adolescents/young adults; for knowledge levels, *p* < 0.001 in school children; *p* < 0.001 in adolescents/young adults). The effect was maintained for 12 months in school children while it decreased in adolescents and young adults with time. Self-esteem significantly increased at 1-month (*p* = 0.002 in school children; *p* = 0.020 in adolescents/young adults) and was maintained for 12 months in both age groups.

**Conclusion:**

The game-based learning programs enhanced health locus of control and self-esteem in childhood cancer survivors. The game-based learning programs could be applied effectively to survivorship care as a new modality of patient education.

**Trial registration:**

This study was retrospectively registered in UMIN-CTR (UMIN000043603) on March 12, 2021.

**Supplementary Information:**

The online version contains supplementary material available at 10.1186/s12885-022-09359-w.

## Background

As the number of childhood cancer survivors (CCSs) continues to grow, reports on cancer-related late effects have been increasing [1–3]. Chronic health conditions associated with cancer therapy include treatment-specific complications such as anthracycline-induced cardiomyopathy, endocrinal disorders and neurocognitive dysfunctions by brain irradiation [4–6]. In addition, CCSs are more susceptible to lifestyle diseases and mental disorders [7, 8].

Nevertheless, it was reported that only 30% of CCSs received survivor-focused care [9]. Many CCSs fail to receive adequate follow-up care for a variety of reasons. Some interrupt their regular visit to long-term follow-up (LTFU) clinics because they are undereducated about potential risks of late effects. Such information has not been given to children before adolescence in Japan. In addition, cancer diagnosis disclosure to children and adolescents was not common before the 1990s when the prognosis of childhood cancer was not yet sufficient and ethics regulation in clinical trials was underdeveloped [10]. In addition, many CCSs interrupt their visits to LTFU clinics around adolescence. CCSs in adolescence are high-risk population with risky health behaviors [11–13] and vulnerable to poor adherence to survivorship care [14]. In Japan, the governmental support for medical expense of childhood cancer care ends at the year of 20, which could trigger the interruption of follow-up care. As chronic health conditions often become apparent clinically after long latency periods, CCSs who lack awareness about their health risks may fail to seek adequate follow-up care. Previous studies identified that CCSs had significant knowledge deficits on their cancer diagnosis and potential risks of late morbidity [15–19]. Therefore, patient education is of great importance to improve their self-awareness for health and adherence to survivorship care.

The Children’s Cancer Group opens educational materials called “Health Link” to the public [20]. Some reports indicated that a variety of educational modalities such as conferences and group-based interventions are beneficial in short-term knowledge attainment [21, 22]. But a questionnaire survey on information needs of CCSs, conducted by the Swiss Childhood Cancer Survivor study, reported that CCSs preferred personalized written materials rather than oral, non-personalized written or online information [23]. These results suggest that personalized risk-profiling and risk-oriented health management should be educated to CCSs. In addition, educational programs should be available for them.

For developing a better educational approach to CCSs, we had an idea on game-based learning programs. We supposed that game-based learning could be a new modality of patient education for CCSs. Computer games are widely accepted by younger generation and can easily simulate potential health events in their future life on the screen. We supposed that game-based learning could match the preference of young CCSs and lead them to learn the importance of survivorship care on their own initiatives. Serious games for education were reported to be effective at engaging children and adolescents in health decision making [24, 25]. Additionally, we considered that CCSs should be provided with continuous education according to their social and intellectual maturation.

Based on these concepts, we developed game-based learning programs, one for school children and another for adolescents and young adults (AYA). In each program, CCSs can play a computer-game with stories constructed along their medical history. The programs are designed to match their developmental stage and to enable continuous approaches to CCSs from school age to AYA by switching from one to another. The acceptability and efficacy of these programs should be evaluated before applying to clinical use by comparing with other modalities of patient education. As the first step, we planned this study to assess the practicability and efficacy of the learning program.

In this pilot study, we requested Japanese CCSs to play the programs for one month and then followed them for one year. This study was to assess the effectiveness of the programs and persistence of the learning effects in a small cohort before implementation of a large scale controlled study. We assessed the effectiveness at 3 domains: health management awareness, self-esteem, and knowledge level gained from the programs. Furthermore, the drop-out rate at each assessment point was measured as an indicator for practicability of this study design.

## Methods

### Computer-based learning tools

Computer-based learning tools were produced to intensify survivorship care in Japan by the LTFU committee of Japan Children’s Cancer Group. The prototypes were made by faculties and students of Future University Hakodate School of Systems Information Sciences and refined under expert guidance by the committee members. The tools are a role-playing game designed for CCSs aged over 10 years and a novel game for AYA. The games are named FUN QUEST and START LINE plus (Additional file 1), respectively. In FUN QUEST, CCSs play the game as the main character after inputting their medical information such as gender, age, diagnosis, and treatment into a computer by a medical staff. The game story is individualized with the information. The player is requested to answer health-related questions on the screen. After each question, the player receives advice on health-related behaviors from other game characters. The game story progresses along the player’s past, current and future life. In START LINE plus, CCSs operate the game as the main player after entering their age, gender, and medical history. As in another game, the game story is individualized with the information. The player learns potential risks of late effects and direction for health management at the major life events such as employment and marriage through virtual experiences. The duration of the game operation is 20-30 min for START LINE plus and 30-50 min for FUN QUEST.

### Subjects

To assess the effectiveness of the game-based learning programs, we recruited CCSs aged over 10 years who regularly visited the LTFU clinic at Mie university hospital, Mie, Japan. A data manager listed potential patients for this study and a pediatric oncologist screened the eligibility. CCSs with severely impaired cognitive function, psychiatric disorder, or developmental disorder were excluded. All subjects were Japanese speaking. They were informed of their cancer diagnosis. The subjects were enrolled into the study during the period between June 2016 and March 2017. They were divided into two groups, school children and AYA, for the respective age-based computer games: FUN QUEST for school-aged children and START LINE for AYA. School children included elementary school children aged between 10 and 12 years and junior high school students aged between 13 and 15 years. AYA were composed of CCSs aged between 16 and 40 years.

Informed consents or assents were obtained from all subjects. Guardians approved participation of their children in the study if the subjects were less than 20 years of age. This study was approved by the institutional review board at Mie University Hospital (No.1533) and registered to the clinical trial register in Japan (UMIN000043603-12/03/2021). This study was conducted in accordance with Ethical Guidelines for Medical and Health Research Involving Human Subjects by the Ministry of Health, Labour and Welfare of Japan.

### Procedure

The background data of the subjects were collected from medical records. The subjects were assessed for their baseline health-management awareness, self-esteem, and knowledge level on cancer-related late effects before the intervention. After the baseline assessment, they were instructed on game operation by an investigator of this study. Each subject was lent a tablet-type device or a laptop computer on which either FUN QUEST for school children or START LINE plus for AYA was installed. The subject was asked to play the game once a week for one month at home. The device was returned at the end of the intervention. Health-management awareness, self-esteem, and knowledge learnt from the game were surveyed with the questionnaire used for the baseline assessment at 1-, 6- and 12-month from the baseline assessment by postal mail.

### Assessment of health-management awareness

The school-life skills scale (SLSS) is commonly used for life-skill assessment at elementary and junior high schools in Japan [26, 27]. The validation has already been carried out in Japanese school children. School-life skills are defined as abilities needed to solve developmental and educational tasks faced by children in their school life. The SLSS-elementary school form contains 43 items in 7 subscales: self-study skills, task completion skills, career decision skills, group activity skills, health maintenance skills, health consultation skills, and peer communication skills. Among those subscales, 7 items in health maintenance and health consultation skills were employed for the assessment of health management awareness of elementary school children (FUN QUEST users) (Additional file 2). The SLSS-junior high school form contains 54 items categorized into 5 subscales: self-study skills, career decision skills, group activity skills, health maintenance skills and peer communication skills. In this study, 9 items in health maintenance skills were used for the assessment of health-management awareness in junior high school students (FUN QUEST users) (Additional file 3). Subjects answered the questions of the selected SLSS by selecting the number that best reflected their perception in a 4-point Likert scale. Definitions for each point value in the scale were as below: 1, strongly disagree; 2, disagree; 3, agree; 4, strongly agree. The result was expressed as the total score of answers to the selected items. A higher score of selected SLSS represented higher health-management awareness. The Japanese version of the perceived health competence scale (PHCS) with 8 question items was used for the assessment of health-management awareness in AYA (START LINE plus users) (Additional file 4). The Japanese version of the scale, which was used in this study, has already been validated [28]. The PHCS was composed of questions with a 5-point Likert scale. Definitions for each point value in the scale were as below: 1, strongly disagree; 2, disagree; 3, neither agree nor disagree; 4, agree; 5, strongly agree. The result was expressed as the total score of all answers. Scoring direction for four negatively worded items was reversed so that a higher score represented higher health management awareness.

### Assessment of self-esteem

We also assessed the effects of the intervention on CCSs’ self-esteem. The Japanese version of the perceived competence scale for children (PCSC) was used for the assessment of self-esteem in school children (FUN QUEST users). The Japanese version of the scale, which was used in this study, has been validated in the Japanese population [29]. The PCSC was composed of 4 subscales: cognitive competence, social competence, physical competence, and general self-worth. In this study, we employed the subscale of general self-worth with 10 question items for assessment of self-esteem in school children (FUN QUEST users) (Additional file 5). The selected items in PCSC were scored on a 4-point Likert scale ranging from 1 (strongly disagree) to 4 (strongly agree). The result was expressed as the total score of answers to the selected items. Four negatively worded items were scored reversely in counting the total score. For AYA (START LINE plus users), the Japanese version of Rosenberg’s self-esteem scale with 10 question items was used (Additional file 6). The scale was confirmed to be reliable and valid in the Japanese population prior to this study [30]. The items were scored on a 4-point Likert scale ranging from 1 (strongly disagree) to 4 (strongly agree). The result was expressed as the total score of all answers. Five negatively worded items were scored reversely in counting the total score.

### Assessment of knowledge levels

To assess the levels of knowledge attained through the game-based learning, we produced written tests named “Knowledge test” from the respective contents of FUN QUEST or START LINE plus (Additional files 7 and 8). Knowledge tests were composed of 6 questions for school children (FUN QUEST users) and 11 for AYA (START LINE plus users). The test required a yes-or-no answer. The results of these two tests were expressed as the total score of right answers, ranging from 0 to 6 or 11, respectively.

### Statistics

For the assessment of health management awareness and self-esteem, the total score of answers was used for statistical analysis. Datasets at the baseline assessment and each of the following points were compared with the Wilcoxon signed-rank test to ascertain improvement after the interventions. Furthermore, datasets at 3 points after the interventions in the subjects completing all 3 post-educational assessments were also compared with the Friedman test to assess persistence of the educational effects. If any significant difference was observed among 3 post-educational assessments, each pair of the 3 assessments were compared with the Wilcoxon signed-rank test.

Similarly, the mean value of the respective knowledge tests was compared between baseline and each of 3 subsequent points with the paired student *t*-test. Persistence of the knowledge level gained with the intervention was analyzed in the subjects completing all 3 post-educational assessments with the One-way repeated measures ANOVA. If any significant difference was observed among 3 post-educational assessments, each pair of the 3 assessments were compared with the paired student *t*-test. All analyses were conducted with two-sided tests at a significance level of 0.05 using IBM SPSS Statistics 24.0 for Windows 10.

## Results

### Subjects

**Fig. 1:**
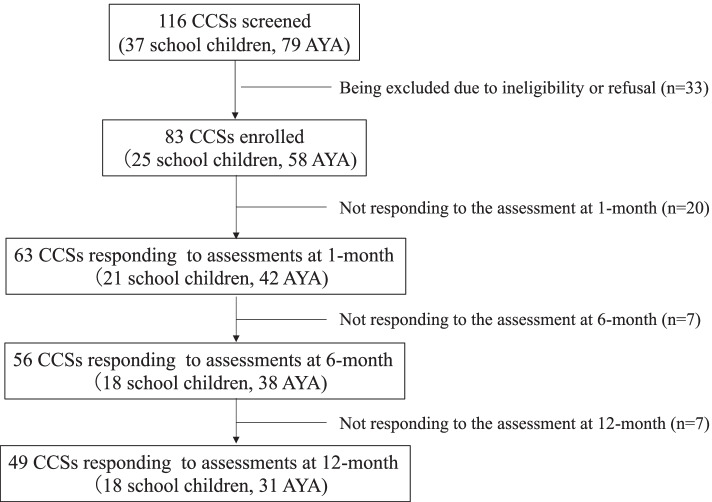
The outline of the study. Subjects were assessed for their health-management awareness, self-esteem, and knowledge level on cancer-related late effects before starting with a game-based learning program. Then they were asked to play an age-matched game once a week for one month at home. Efficacy of the intervention was surveyed with questionnaires at 1-, 6- and 12-month from the baseline assessment. CCSs, childhood cancer survivors; AYA, adolescents and young adults

The outline of subject recruitment in this study was indicated in Fig. 1. Eighty-three CCSs participated in the study. Among 83 participants, 25 school children played FAN QUEST and 58 AYA, START LINE plus. Male ratio in FUN QUEST users was higher than that in START LINE plus users (64.0 vs. 51.7%). Leukemia/Lymphoma was the most frequent diagnosis in both cohorts (56.0% in FUN QUEST and 65.5% in START LINE plus users). All subjects received at least one treatment modality, consisting of chemotherapy, radiation, hematopoietic stem cell transplantation or surgery.

Among 83 subjects, 20 participants withdrew from the study at 1-month assessment, 7 did not respond to the 6-month survey, and another 7 had no response to the 12-month survey (Additional file 9). The characteristics of 63 subjects who completed 1^st^ follow-up assessment at 1-month was shown in Table 1. The mean age at diagnosis of the primary cancer was 7.1 years (range, 1-12) in FUN QUEST users and 8.2 years (range, 0-19) in START LINE plus users. The mean age at the baseline survey was 12.4 years (range, 10-15) in FUN QUEST users and 22.7 years (range, 16-32) in START LINE plus users.

**Table 1:** Characteristics of the subjects

	Entire Cohort n (%)	FUN QUEST n (%)	START LINE plus n (%)
Gender			
Male	37 (58.7)	14 (66.7)	23 (54.8)
Female	26 (41.3)	7 (33.3)	19 (45.2)
Diagnosis			
Leukemia	33 (52.4)	9 (42.9)	24 (57.1)
Lymphoma	6 (9.5)	2 (9.5)	4 (9.5)
Brain tumor	7 (11.1)	4 (19.0)	3 (7.1)
Solid tumor	17 (27.0)	6 (28.6)	11 (26.2)
Treatment			
Chemotherapy	61 (96.8)	19 (90.5)	42(100.0)
Radiation	31 (49.2)	9 (49.2)	12 (28.6)
Hematopoietic stem cell transplantation	6 (9.5)	2 (9.5)	4 (9.5)
Surgery	18 (28.6)	6 (28.6)	12(28.6)
Age at diagnosis			
Mean	7.86	7.10	8.24
Range	0-19	1﻿﻿-12	0﻿-19
SD	4.72	3.25	5.26
Age at the baseline assessment			
Mean	19.25	12.43	22.67
Range	10﻿-32	10﻿﻿-15	16﻿-32
SD	6.17	1.53	4.58

The characteristics of 63 subjects who completed 1^st^ follow-up assessment at 1-month were indicated

A total of 34 (41.0%) subjects withdrew from the study over the period of 12 months. Female subjects withdrew more than male subjects (48.6 vs. 34.8%). Among diseases categories, CCSs with brain tumor showed a higher non-withdrawal rate. There were some negative comments from the withdrawals such as “boring” and “laborious setting manipulation”.

The numbers of responders were 63 at 1-month, 56 at 6-month, and 49 at 12-month. Forty-nine (59.0%) who completed the entire course of the surveys were applied for the analysis on persistence of the learning effects.

### Health management awareness

Changes from the baseline to all 3 assessment points were indicated in Additional file 10. At 1-month, 11 elementary school children, 10 junior high school students, and 42 AYA were assessed. A significant increase of the score was observed in all age categories (*p* = 0.008 in elementary school children; *p* = 0.003 in junior high school students; *p* < 0.001 in AYA). The change of the mean score from the baseline was from 20.09 to 25.73 in elementary school children, from 23.30 to 31.00 in junior high school students, and from 24.55 to 31.21 in AYA. At 6-month, 9 elementary school children, 9 junior high school students, and 38 AYA responded. At 12-month, 9 elementary school children, 9 junior high school students, and 31 AYA were assessed. Similarly, the scores at 6- and 12-month assessments were significantly higher than that at the baseline in any age group(*p* = 0.008 in elementary school children at 6-month; *p* = 0.007 in junior high school students at 6-month; *p* = 0.002 in AYA at 6-month; *p* = 0.008 in elementary school children at 12-month; *p* = 0.007 in junior high school students at 12-month; *p* = 0.001 in AYA at 12-month). The changes of the mean score from the baseline to 6- and 12-month in elementary school children were from 19.56 to 25.11 and from 19.56 to 25.44, respectively while those in junior high school students were from 22.44 to 29.00 and from 22.44 to 30.44. Those in AYA were from 24.00 to 28.45 and from 23.48 to 28.48. The trend of these findings was similarly observed in leukemia/lymphoma, solid tumor and brain tumor groups.

### Self-esteem

Changes from the baseline to all 3 assessment points were indicated in Additional file 10. The responders were 21 school children and 42 AYA at 1-month, 18 school children and 38 AYA at 6-month, and 18 school children and 31 AYA at 12-month. The score at 1-month after the intervention was significantly higher than that at the baseline in both school children and AYA (*p* = 0.003 in school children; *p* = 0.004 in AYA). Similarly, the score at 12-month in AYA was significantly higher than that at the baseline (*p* = 0.021). But the scores at 6- and 12-month in school children and at 6-month in AYA were not significantly higher as compared to those at the baseline. The changes of the mean score from the baseline to 1-, 6- and 12-month in school children was from 29.24 to 33.00, from 28.94 to 31.50, and from 28.94 to 30.50, respectively. Those in AYA were from 34.50 to 36.88, from 34.47 to 34.71, and from 34.32 to 38.35. The trend of these findings was similarly observed in leukemia/lymphoma, solid tumor and brain tumor groups.

### Knowledge levels

The responders were 21 school children and 42 AYA at 1-month, 18 school children and 38 AYA at 6-month, and 18 school children and 31 AYA at 12-month.

The scores at 1-, 6- and12-month after the intervention were significantly higher than that at the baseline in school children. The increase was more significant at 1-month than 6- and 12-month (*p* < 0.001 at 1-month; *p* = 0.001 at 6-month; *p* = 0.003 at 12-month) (Additional file 10). Those were also significantly higher in AYA (*p* < 0.001 at any point). The changes of the mean score from baseline to 1-, 6- and 12-month in school children was from 2.00 to 5.00, from 3.00 to 4.00, and from 3.00 to 5.00, respectively. Those in AYA was from 7.50 to 11.00, from 7.00 to 10.00, and from 7.0 to 9.00. The trend of these findings was similarly observed in leukemia/lymphoma, solid tumor and brain tumor groups.

### Persistence of learning effects during the first 12 months

**Fig. 2:**
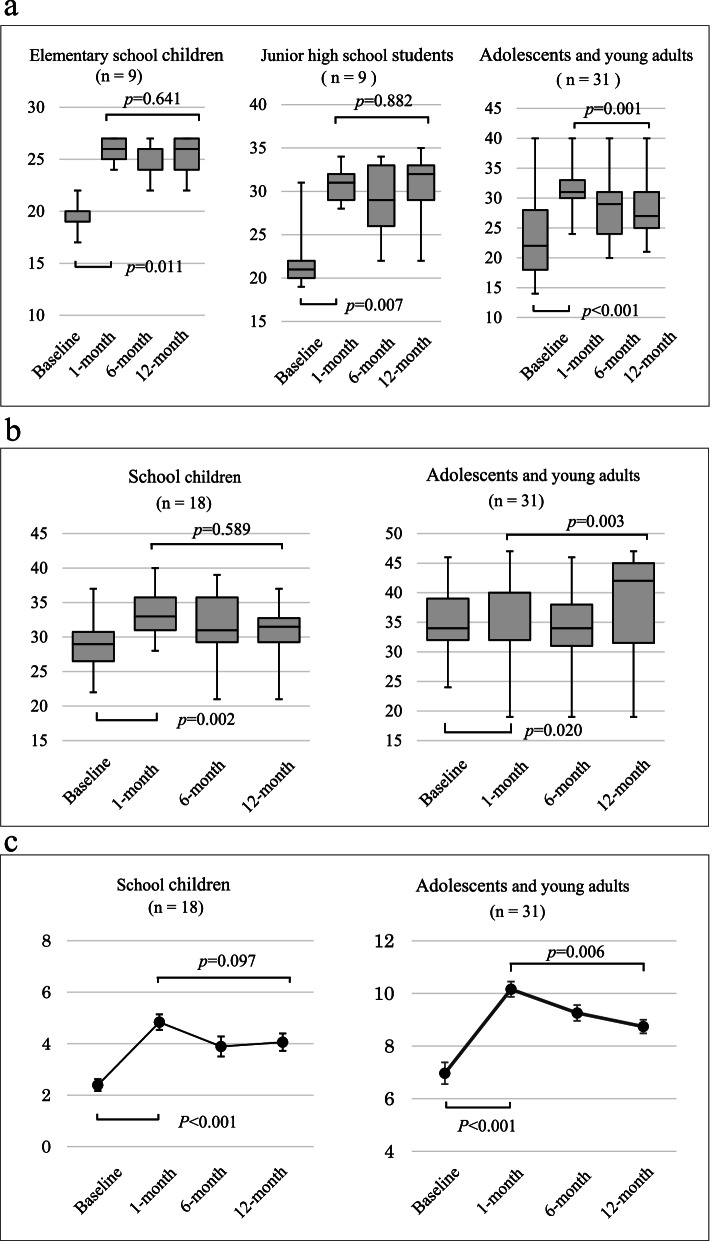
Persistence of learning effects in subject completing all assessments. **a**, health management awareness; **b**, self-esteem; **c**, knowledge level. Distributions of the total scores of surveys for health management awareness and self-esteem in each age category are shown as box plots. For knowledge level, mean scores ± SE of Knowledge tests at two age categories are shown. First, we analyzed an increase in the score at 1-month from the baseline with the Wilcoxon signed-rank test or the paired student *t*-test. Then we evaluated the dataset at 3 post-intervention assessment points with the Friedman test or the One-way repeated measures ANOVA

Health management awareness at 1-month was significantly higher than that at the baseline in any age group (*p* = 0.011 in elementary school children; *p* = 0.007 in junior high school students; *p* < 0.001 in AYA) (Fig. 2a). The change of the mean score from the baseline to 1-month was from 19.56 to 25.78 in elementary school children, from 22.44 to 30.67 in junior high school students, and from 23.48 to 31.90 in AYA. Health management awareness in school children did not significantly decrease during the first 12 months (*p* = 0.641 in elementary school children; *p* = 0.882 in junior high school students) while it significantly decreased in AYA as time passed (*p* = 0.001). The significant decrease was noted between 1-month and 6-month (*p* = 0.001) and between 1-month and 12-month (*p* = 0.002).

The mean scores in elementary school children were 25.78 at 1-month, 25.11 at 6-month, and 25.44 at 12-month while those in junior high school students were 30.67 at 1-month, 29.00 at 6-month, and 30.44 at 12-month. Those in AYA were 31.90 at 1-month, 28.26 at 6-month, and 28.48 at 12-month. Self-esteem significantly increased at 1-month (*p* = 0.002 in school children; *p* = 0.020 in AYA) as compared to that at the baseline. The changes of the mean score were from 28.94 to 33.28 in school children and from 34.32 to 36.23 in AYA. Self-esteem did not significantly decrease during the first 12 months in school children (*p* = 0.589) (Fig. 2b). The mean scores at each point in school children were 33.28 at 1-month, 31.50 at 6-month, and 30.50 at 12-month.

In AYA, self-esteem significantly increased (*p* = 0.003). The increase was significant between 6- and 12-month (*p* = 0.004). The mean scores at each point in AYA were 36.23 at 1-month, 34.42 at 6-month, and 38.35 at 12-month.

The score of respective Knowledge tests significantly increased at 1-month in both age groups compared to each baseline value (*p* < 0.001 in both groups) (Fig. 2c). The changes of the mean score were from 3.00 to 5.00 in school children and from 7.00 to 11.00 in AYA. The effect did not decrease in school children during the first 12 months (*p* = 0.097) while it significantly decreased in AYA (*p* = 0.006). The decrease from 1-month assessment is significant at both 6- and 12-month assessments (*p* = 0.010 at 6-month, *p* = 0.009 at 12-month). The mean scores at each point were 5.00 at 1-month, 4.00 at 6-month, and 5.00 at 12-month in school children and 11.00 at 1-month, 9.00 at 6-month, and 9.00 at 12-month in AYA.

## Discussion

We developed the game-based learning programs for CCSs and conducted the questionnaire surveys for one year to assess the effectiveness of the programs and persistence of the learning effects. We assessed the effectiveness at 3 domains: health management awareness, self-esteem, and knowledge level gained from the programs. Health management awareness is critical to ensure survivors’ engagement in health promotion, early detection and timely intervention for cancer-related late effects. Self-esteem was assessed because recognizing the potential risks may have negative impact on their psychosocial conditions. It was also expected that self-esteem may be enhanced by attaining skills for problem-solving. We also focused on knowledge levels obtained with the intervention. The intervention raised both health management awareness and knowledge levels at each age category. The effects were maintained for one year after the intervention in school children but decreased in AYA with time after the intervention. The results in school children coincided with the observation in a previous study among asthmatic children aged between 9 and 13 years who were educated with a video-game designed to facilitate self-management [31]. In that study, the educated cohort had a higher level of knowledge about self-regulation, treatment and prevention as compared to the group without educational intervention. However, in this study the similar result was not observed in AYA. Some additional educational approaches may be needed for AYA to prevent declines in the effects.

Both school children and AYA showed significantly higher self-esteem at 1-month as compared to that at baseline and maintained the effect for one year. The high self-esteem is expected to guide CCSs toward behavioral change to achieve better quality of life. A previous report indicated that self-esteem was an important predictor for social domain of quality of life [32].

From these findings, we can say that the game-based learning programs could be a new modality of patient education in cancer survivorship care. Educational tools using multimedia have already been introduced to patient education for children with chronic diseases [33, 34]. A study revealed that educational video games for patients with diabetes could improve their diabetes-related self-efficacy, communication with parents about diabetes, and self-care behaviors [33]. Another study targeting teens with solid tumor demonstrated that teaching with a multimedia CD-ROM increased their internal locus of control more effectively than the intervention with a handbook [34].

In the field of pediatric oncology, patient education using multimedia was introduced mainly for improving their adherence to medication and supporting their understanding of diagnosis [34–36]. The game-based learning programs for CCSs are unique. Some educational websites dealing with late effects are available. However, the number is very few, and the websites are not interactive [37–39]. A systematic literature review published in 2018 identified 14 pediatric cancer websites, none of which focused exclusively on late effects [40]. Another report described that learning resources using a website may not well function due to user’s low motivation to access [41]. The game tools may have advantages in educating young people because of their preferences for playing games. Computer games are also superior in high interactivity and capability to create attractive graphics.

The game-based learning programs could be effectively applied to the practice of patient education for CCSs. High awareness of health management based on understanding of potential risks of late morbidity may facilitate them to adhere to adequate survivorship care. The continuous approach from childhood to adolescence and young adulthood is expected to improve the effects of patient education. Additionally, the programs may function as an educational modality complementary to standard approaches such as physician’s counseling.

The findings in this study need to be considered in the context of its limitations. Although the subjects were instructed to play once a week, the actual frequency of playing the games was not recorded. It was possible that they played more or less than instructed, which may have impacted the outcomes. Considerably large number of subjects withdrew from the study during 1-year follow-up. The high withdrawal rate may be partially due to the long study period and the survey method which requested the subjects to send their completed questionnaires back by postal mail. In the withdrawals, there were more females than males. The results could indicate less preference to game playing in female subjects. Among diseases categories, CCSs with brain tumor showed a higher non-withdrawal rate. CCSs with brain tumor are at higher risks of late effects. Therefore, they may have high awareness for learning their health. The high withdrawal rate might affect the results because the subjects highly adherent to the study protocol might have higher awareness on health management. The backgrounds of withdrawals should be examined in detail to create better contents of the program. Furthermore, this study was not designed as a randomized control study because this study aimed to evaluate the practicability and efficacy of the game-based programs. The results proved that the programs were applicable to patient education for CCSs regularly accessing LTFU care and warranted further studies to compare the game-based learning programs with other ordinary modalities in a controlled study. The effects of the programs on CCSs who do not regularly visit the LTFU clinic should be evaluated in the next study.

## Conclusions

We produced game-based self-learning programs for CCSs in a project to enhance survivorship care in Japan. The game-based programs were designed to provide essential information on cancer-related late effects to CCSs. This study showed that the programs improved their health management awareness, knowledge levels on late effects, and self-esteem in a cohort of CCSs regularly visiting LTFU clinic. The study also revealed that the persistence of health management awareness and knowledge level decreased with time in AYA.

The dataset supporting the conclusions of this article is included within the article and Additional file 11.

## Supplementary Information

**Additional file 1.** Game-based learning tools for school children, FUN QUEST and for adolescents and adults, START LINE plus We developed two type of computer-based game tools. One is a role-playing game for school children, named FUN QUEST. Another is a novel game for adolescents and young adults, named START LINE plus. The games are configured with Japanese language but can be applied to multilingual settings. The system requirement is Microsoft Windows OS with Adobe Flash Player or Apple iPad. The media play at 1.5x speed in this file.

**Additional file 2.** The school-life skills scale - elementary school form, items selected for this study.

**Additional file 3.** The school-life skills scale - junior high school form, items selected for this study.

**Additional file 4.** The perceived health competence scale.

**Additional file 5.** The perceived competence scale for children, items selected for this study.

**Additional file 6.** Rosenberg’s self-esteem scale.

**Additional file 7.** Knowledge test for FUN QUEST users.

**Additional file 8.** Knowledge test for START LINE plus users.

**Additional file 9.** Characteristics of the withdrawals.

**Additional file 10.** Change of health management awareness, self-esteem, and knowledge level from the baseline.

**Additional file 11.** Dataset.

## Data Availability

All data generated or analyzed during this study are included in this published article and its supplementary information files.

## Abbreviations

CCSs: Childhood cancer survivors
LTFU: Long-term follow-up
AYA: Adolescents and young adults
SLSS: The school-life skills scale
PHCS: The perceived health competence scale
PCSC: The perceived competence scale for children
