# Supplementary material for: A pilot study of game-based learning programs for childhood cancer survivors
Source: BMC Cancer. 2022 Mar 29;22:340. doi: 10.1186/s12885-022-09359-w (PMC8962149; doi:10.1186/s12885-022-09359-w)
Supplement: Supplementary file 3 — Additional file 3. The school-life skills scale - junior high school form, items selected for this study. [file 12885_2022_9359_MOESM3_ESM.docx]

**Additional File 3** The school-life skills scale - junior high school form, items selected for this study

Q1. When you feel sick, you can talk it to someone in words of your own.

Q2. When you feel sick, you can ask some adult for his/her advice on your health condition without leaving it as it is.

Q3. When you get tired, you can have enough rest.

Q4. You can talk about your worries on changes of physical conditions with someone.

Q5. You can take care to have enough sleep to maintain a life rhythm.

Q6. You can have nutritionally well-balanced diet for your health.

Q7. You can convey your opinion or idea to your parents firmly.

Q8. You can convey your opinion or idea to your schoolteachers.

Q9. You have several ways of relaxing your mind and body.
