# Supplementary material for: A pilot study of game-based learning programs for childhood cancer survivors
Source: BMC Cancer. 2022 Mar 29;22:340. doi: 10.1186/s12885-022-09359-w (PMC8962149; doi:10.1186/s12885-022-09359-w)
Supplement: Supplementary file 4 — Additional file 4. The perceived health competence scale. [file 12885_2022_9359_MOESM4_ESM.docx]

**Additional File 4** The perceived health competence scale

Q1. I handle myself well with respect to my health.

Q2. No matter how hard I try, my health just doesn’t turn out the way I would like.

Q3. It is difficult for me to find effective solutions to the health problems that come my way.

Q4. I succeed in the projects I undertake to improve my health.

Q5. I’m generally able to accomplish my goals with respect to my health.

Q6. I find my efforts to change things I don’t like about my health are ineffective.

Q7. Typically, my plans for my health don’t work out well.

Q8. I am able to do things for my health as well as most other people.

Four negatively worded questions (Q2, 3, 6, 7) were scored reversely in counting the total score.
