# Supplementary material for: A pilot study of game-based learning programs for childhood cancer survivors
Source: BMC Cancer. 2022 Mar 29;22:340. doi: 10.1186/s12885-022-09359-w (PMC8962149; doi:10.1186/s12885-022-09359-w)
Supplement: Supplementary file 5 — Additional file 5. The perceived competence scale for children, items selected for this study. [file 12885_2022_9359_MOESM5_ESM.docx]

**Additional File 5** The perceived competence scale for children, items selected for this study

Q1. You have self-confidence .

Q2. You can handle a large part of your task better than others.

Q3. You have much to be proud of.

Q4.You feel that nothing goes well with you.

Q5. You are content with what you are now.

Q6. You can surely imagine that you will be an achiever of success.

Q7. You are a person of no worth or use.

Q8. You can express your opinion with confidence in yourself.

Q9. You don’t have good points so much.

Q10. You always worry about making mistakes.

Four negatively worded questions (Q4, 7, 9, 10) were scored reversely in counting the total score.
