# Supplementary material for: A pilot study of game-based learning programs for childhood cancer survivors
Source: BMC Cancer. 2022 Mar 29;22:340. doi: 10.1186/s12885-022-09359-w (PMC8962149; doi:10.1186/s12885-022-09359-w)
Supplement: Supplementary file 6 — Additional file 6. Rosenberg’s self-esteem scale. [file 12885_2022_9359_MOESM6_ESM.docx]

**Additional File 6** Rosenberg’s self-esteem scale

Q1. I feel that I'm a person of worth, at least on an equal plane with others.

Q2. I feel that I have a number of good qualities.

Q3. All in all, I am inclined to feel that I am a failure.

Q4. I am able to do things as well as most other people.

Q5. I feel I do not have much to be proud of.

Q6. I take a positive attitude toward myself.

Q7. On the whole, I am satisfied with myself.

Q8. I wish I could have more respect for myself.

Q9. At times I think I am no good at all.

Q10. I certainly feel useless at times.

Five negatively worded questions (Q3, 5, 8, 9, 10) were scored reversely in counting the total score.
