# Supplementary material for: A pilot study of game-based learning programs for childhood cancer survivors
Source: BMC Cancer. 2022 Mar 29;22:340. doi: 10.1186/s12885-022-09359-w (PMC8962149; doi:10.1186/s12885-022-09359-w)
Supplement: Supplementary file 7 — Additional file 7. Knowledge test for FUN QUEST users. [file 12885_2022_9359_MOESM7_ESM.docx]

**Additional File 7** Knowledge test for FUN QUEST users

Q1. Late effects mean health issues arising secondarily from cancer in an early age and its treatment.

Q2. Late effects may influence on various aspects of your health conditions.

Q3. You do not need to mind late effects if you once recover your health after cancer treatment.

Q4. You have to visit a long-term follow-up clinic monthly from now on.

Q5. Physical examination by a physician is the only purpose for long-term follow-up.

Q6. You can ask medical staff for their advices on schooling, employment, personal relationship and marriage.
