# Supplementary material for: A pilot study of game-based learning programs for childhood cancer survivors
Source: BMC Cancer. 2022 Mar 29;22:340. doi: 10.1186/s12885-022-09359-w (PMC8962149; doi:10.1186/s12885-022-09359-w)
Supplement: Supplementary file 8 — Additional file 8. Knowledge test for START LINE plus users. [file 12885_2022_9359_MOESM8_ESM.docx]

**Additional File 8** Knowledge test for START LINE plus users

Q1. Late effects mean health issues remaining after cancer treatment or emerging after the treatment.

Q2. Late effects are usually treatment-specific.

Q3. Early intervention is not necessary for late effects.

Q4. Smoking could be a risk factor for late effects.

Q5. Knowing your own illness is beneficial for early detection of the late effects.

Q6. Increased risk for congenital anomaly or cancer in offspring of childhood cancer survivors is not proved except for special cases.

Q7. You should eat your favorite foods as much as you want if your health is well.

Q8. You have to visit a long-term follow-up clinic monthly from now on even after the completion of cancer treatment.

Q9. You are examined for laboratory tests at a long-term follow-up clinic based on the treatment which you received.

Q10. You should not ask medical staff for advices on your privacy such as schooling, employment, personal relationship and marriage.

Q11. It is recommended that you should consult your fertility because the fertility of childhood cancer survivors could be affected by cancer treatment.
