# Supplementary material for: A pilot study of game-based learning programs for childhood cancer survivors
Source: BMC Cancer. 2022 Mar 29;22:340. doi: 10.1186/s12885-022-09359-w (PMC8962149; doi:10.1186/s12885-022-09359-w)
Supplement: Supplementary file 10 — Additional file 10. Change of health management awareness, self-esteem, and knowledge level from the baseline. [file 12885_2022_9359_MOESM10_ESM.pptx]

## Slide 1
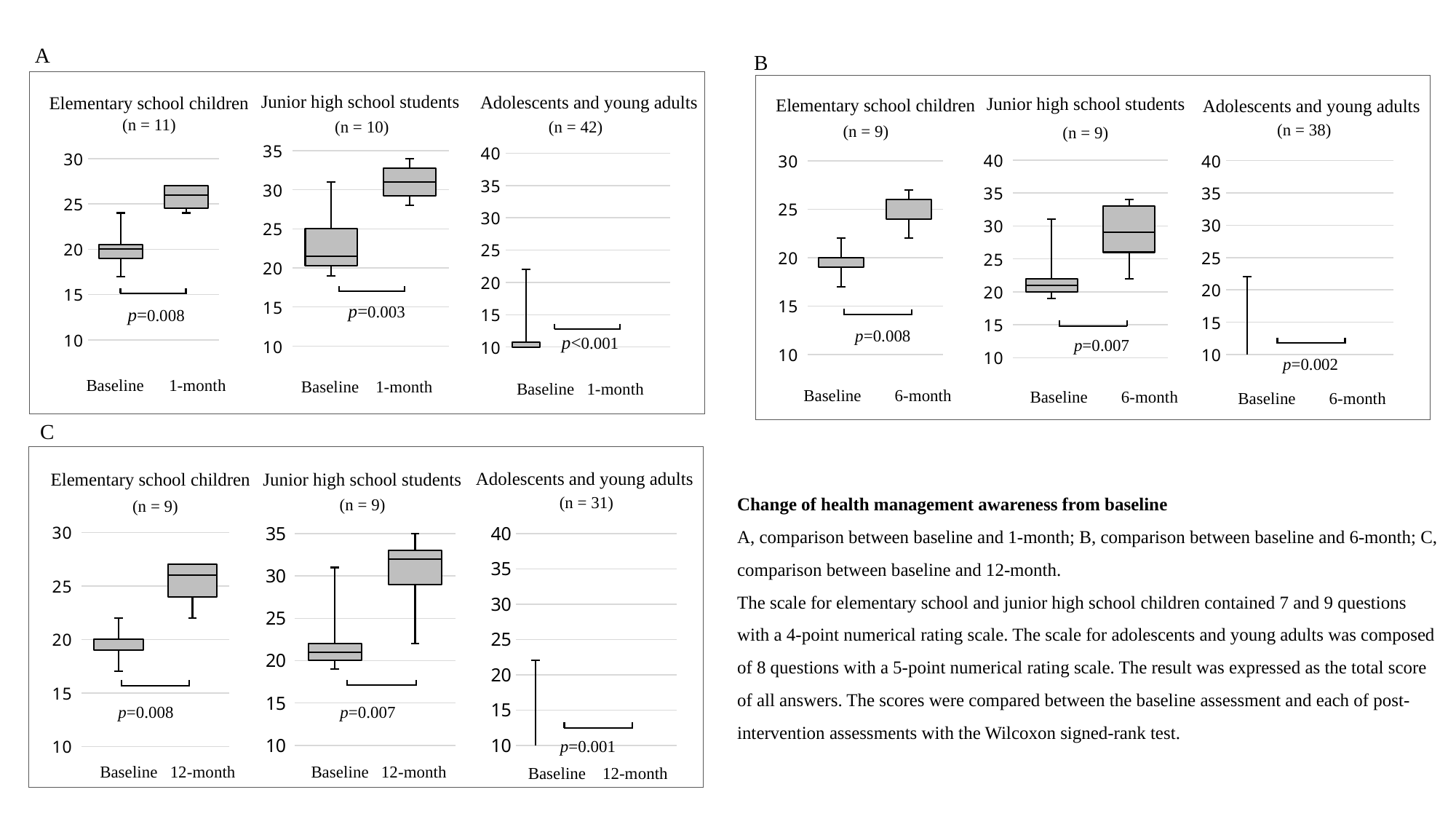

A
Junior high school students
Adolescents and young adults
Elementary school children
(n = 11)
(n = 10)
(n = 42)
### Chart
| Category | 25% | 中央値-25% | 75%-中央値 |
|---|---|---|---|
| Baseline | 20.25 | 1.25 | 3.5 |
| 1 month | 29.25 | 1.75 | 1.75 |
### Chart
| Category | 25% | 中央値-25% | 75%-中央値 |
|---|---|---|---|
| Baseline | 18.0 | 7.0 | 3.75 |
| 1 month | 28.0 | 3.0 | 1.0 |
### Chart
| Category | 25% | 中央値-25% | 75%-中央値 |
|---|---|---|---|
| Baseline | 19.0 | 1.0 | 0.5 |
| 1 month | 24.5 | 1.5 | 1.0 |p=0.003
p=0.008
p<0.001
Baseline 1-month
Baseline 1-month
Baseline 1-month
B
Junior high school students
Elementary school children
Adolescents and young adults
(n = 38)
(n = 9)
(n = 9)
### Chart
| Category | 25% | 中央値-25% | 75%-中央値 |
|---|---|---|---|
| Baseline | 20.0 | 1.0 | 1.0 |
| 6 months | 26.0 | 3.0 | 4.0 |
### Chart
| Category | 25% | 中央値-25% | 75%-中央値 |
|---|---|---|---|
| Baseline | 18.0 | 6.5 | 3.5 |
| 6 months | 24.0 | 5.5 | 1.5 |
### Chart
| Category | 25% | 中央値-25% | 75%-中央値 |
|---|---|---|---|
| Baseline | 19.0 | 1.0 | 0.0 |
| 6 months | 24.0 | 2.0 | 0.0 |p=0.008
p=0.007
p=0.002
Baseline 6-month
Baseline 6-month
Baseline 6-month
 C
Adolescents and young adults
Junior high school students
Elementary school children
(n = 31)
(n = 9)
(n = 9)
### Chart
| Category | 25% | 中央値-25% | 75%-中央値 |
|---|---|---|---|
| Baseline | 19.0 | 1.0 | 0.0 |
| 12 months | 24.0 | 2.0 | 1.0 |
### Chart
| Category | 25% | 中央値-25% | 75%-中央値 |
|---|---|---|---|
| Baseline | 20.0 | 1.0 | 1.0 |
| 12 months | 29.0 | 3.0 | 1.0 |
### Chart
| Category | 25% | 中央値-25% | 75%-中央値 |
|---|---|---|---|
| Baseline | 18.0 | 4.0 | 6.0 |
| 12 months | 25.0 | 2.0 | 4.0 |p=0.008
p=0.007
p=0.001
Baseline 12-month
Baseline 12-month
Baseline 12-month
Change of health management awareness from baseline
A, comparison between baseline and 1-month; B, comparison between baseline and 6-month; C, comparison between baseline and 12-month.
The scale for elementary school and junior high school children contained 7 and 9 questions with a 4-point numerical rating scale. The scale for adolescents and young adults was composed of 8 questions with a 5-point numerical rating scale. The result was expressed as the total score of all answers. The scores were compared between the baseline assessment and each of post-intervention assessments with the Wilcoxon signed-rank test.

## Slide 2
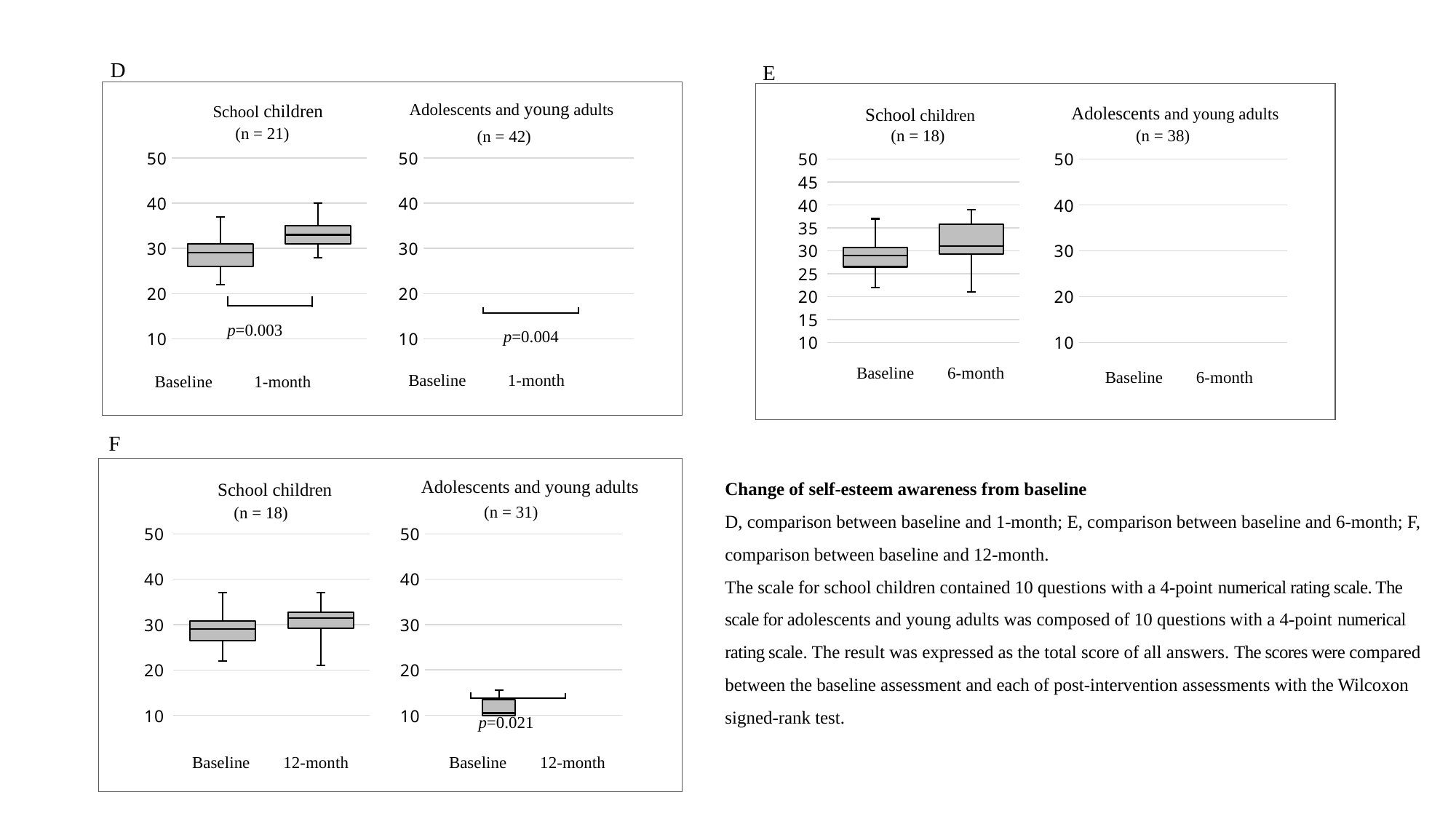

D
Adolescents and young adults
School children
(n = 21)
(n = 42)
### Chart
| Category | 25% | 中央値-25% | 75%-中央値 |
|---|---|---|---|
| Baseline | 26.0 | 3.0 | 2.0 |
| 1 month | 31.0 | 2.0 | 2.0 |
### Chart
| Category | 25% | 中央値-25% | 75%-中央値 |
|---|---|---|---|
| Baseline | 32.0 | 1.5 | 4.25 |
| 1 month | 32.5 | 7.0 | 0.5 |p=0.003
p=0.004
Baseline 1-month
Baseline 1-month
E
Adolescents and young adults
School children
(n = 18)
(n = 38)
### Chart
| Category | 25% | 中央値-25% | 75%-中央値 |
|---|---|---|---|
| Baseline | 26.5 | 2.5 | 1.75 |
| 6 months | 29.25 | 1.75 | 4.75 |
### Chart
| Category | 25% | 中央値-25% | 75%-中央値 |
|---|---|---|---|
| Baseline | 32.0 | 2.0 | 3.75 |
| 6 months | 31.0 | 3.0 | 4.75 |Baseline 6-month
Baseline 6-month
F
Adolescents and young adults
School children
(n = 31)
(n = 18)
### Chart
| Category | 25% | 中央値-25% | 75%-中央値 |
|---|---|---|---|
| Baseline | 32.0 | 2.0 | 5.0 |
| 12 months | 31.5 | 10.5 | 3.0 |
### Chart
| Category | 25% | 中央値-25% | 75%-中央値 |
|---|---|---|---|
| Baseline | 26.5 | 2.5 | 1.75 |
| 12 months | 29.25 | 2.25 | 1.25 |p=0.021
Baseline 12-month
Baseline 12-month
Change of self-esteem awareness from baseline
D, comparison between baseline and 1-month; E, comparison between baseline and 6-month; F, comparison between baseline and 12-month.
The scale for school children contained 10 questions with a 4-point numerical rating scale. The scale for adolescents and young adults was composed of 10 questions with a 4-point numerical rating scale. The result was expressed as the total score of all answers. The scores were compared between the baseline assessment and each of post-intervention assessments with the Wilcoxon signed-rank test.

## Slide 3
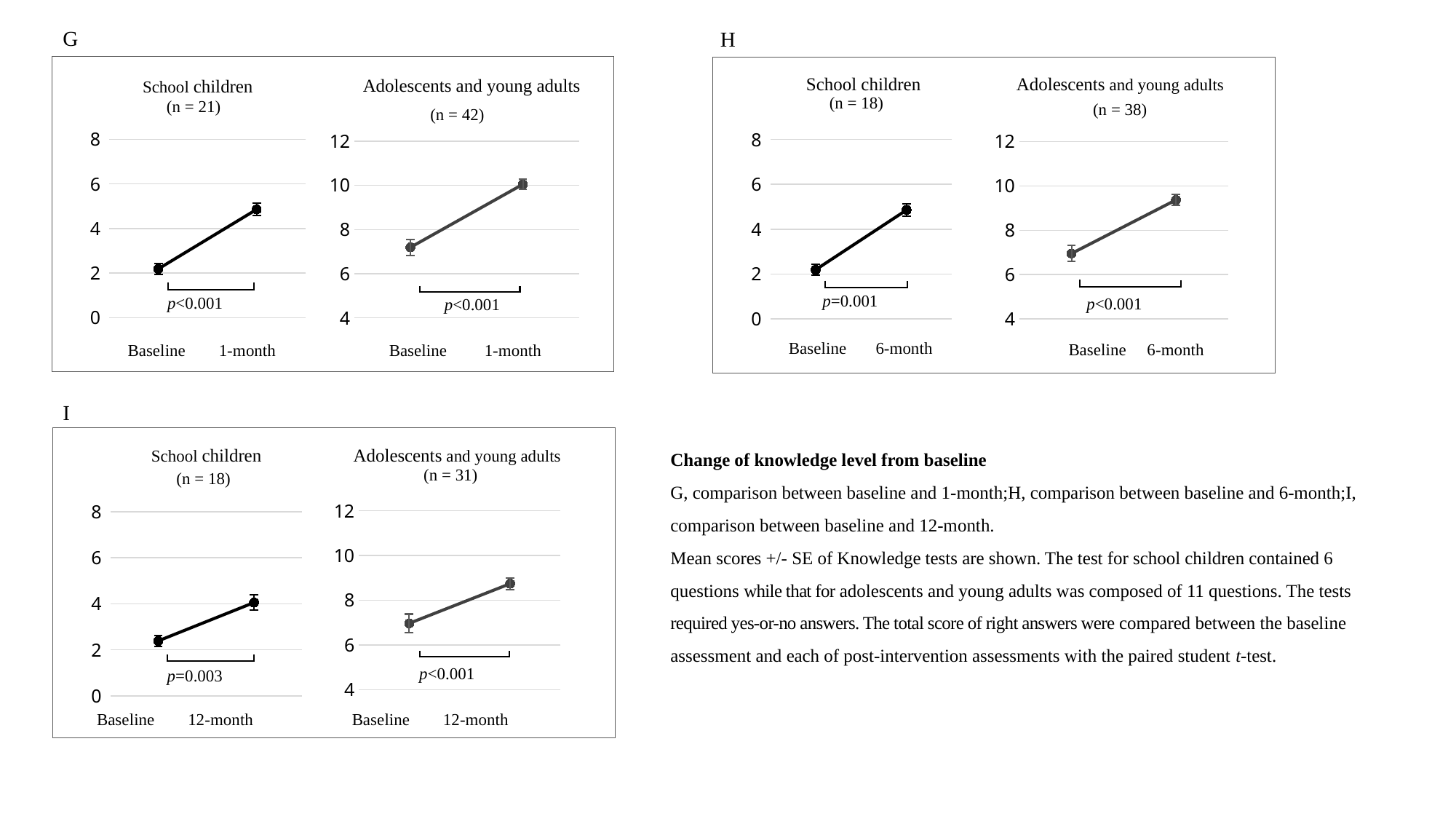

G
H
Adolescents and young adults
School children
Adolescents and young adults
School children
(n = 18)
(n = 21)
(n = 38)
(n = 42)
### Chart
| Category | |
|---|---|
| Baseline | 2.1904761904761907 |
| 1 month | 4.857142857142857 |
### Chart
| Category | |
|---|---|
| Baseline | 2.1904761904761907 |
| 1 month | 4.857142857142857 |
### Chart
| Category | |
|---|---|
| Baseline | 7.190476190476191 |
| 1 month | 10.047619047619047 |
### Chart
| Category | |
|---|---|
| Baseline | 6.947368421052632 |
| 6 months | 9.368421052631579 |p=0.001
p<0.001
p<0.001
p<0.001
Baseline 6-month
Baseline 6-month
Baseline 1-month
Baseline 1-month
I
Change of knowledge level from baseline
G, comparison between baseline and 1-month;H, comparison between baseline and 6-month;I, comparison between baseline and 12-month.
Mean scores +/- SE of Knowledge tests are shown. The test for school children contained 6 questions while that for adolescents and young adults was composed of 11 questions. The tests required yes-or-no answers. The total score of right answers were compared between the baseline assessment and each of post-intervention assessments with the paired student t-test.
Adolescents and young adults
School children
(n = 31)
(n = 18)
### Chart
| Category | |
|---|---|
| Baseline | 6.967741935483871 |
| 12 months | 8.741935483870968 |
### Chart
| Category | |
|---|---|
| Baseline | 2.388888888888889 |
| 12 months | 4.055555555555555 |p<0.001
p=0.003
Baseline 12-month
Baseline 12-month
